# Factors affecting two-point discrimination in Argus II patients

**DOI:** 10.3389/fnins.2022.901337

**Published:** 2022-08-24

**Authors:** Ezgi I. Yücel, Roksana Sadeghi, Arathy Kartha, Sandra Rocio Montezuma, Gislin Dagnelie, Ariel Rokem, Geoffrey M. Boynton, Ione Fine, Michael Beyeler

**Affiliations:** ^1^Department of Psychology, University of Washington, Seattle, WA, United States; ^2^Department of Biomedical Engineering, Johns Hopkins School of Medicine, Baltimore, MD, United States; ^3^Department of Ophthalmology, Johns Hopkins School of Medicine, Baltimore, MD, United States; ^4^Department of Ophthalmology and Visual Neurosciences, University of Minnesota, Minneapolis, MN, United States; ^5^eScience Institute, University of Washington, Seattle, WA, United States; ^6^Department of Computer Science, University of California, Santa Barbara, Santa Barbara, CA, United States; ^7^Department of Psychological and Brain Sciences, University of California, Santa Barbara, Santa Barbara, CA, United States

**Keywords:** sight restoration, retinal prosthesis, retina, current spread, modeling, psychophysics

## Abstract

Two of the main obstacles to the development of epiretinal prosthesis technology are electrodes that require current amplitudes above safety limits to reliably elicit percepts, and a failure to consistently elicit pattern vision. Here, we explored the causes of high current amplitude thresholds and poor spatial resolution within the Argus II epiretinal implant. We measured current amplitude thresholds and two-point discrimination (the ability to determine whether one or two electrodes had been stimulated) in 3 blind participants implanted with Argus II devices. Our data and simulations show that axonal stimulation, lift and retinal damage all play a role in reducing performance in the Argus 2, by either limiting sensitivity and/or reducing spatial resolution. Understanding the relative role of these various factors will be critical for developing and surgically implanting devices that can successfully subserve pattern vision.

## Introduction

Diseases such as Retinitis Pigmentosa (RP) and Age-Related Macular Degeneration (AMD) cause photoreceptor degeneration that results in severe loss of vision at later stages (Pfeiffer et al., 2020). RP affects approximately 1/4,000 (Ayuso and Millan, 2010) and late AMD affects 1/300 adults globally (Wong et al., 2014). While some of these diseases have treatments that slow progression (Mitchell et al., 2018), none are curable. Once the disease has progressed to severe vision loss, one of the few potential treatments is implantation with a retinal or cortical prosthesis. Based on a principle similar to cochlear implants, retinal implants use an array of electrodes to stimulate remaining (non-photoreceptor) neurons in the retina to evoke phosphenes. The Argus II (Second Sight Medical Products, Inc.) is one of two FDA-approved devices, with the other being a suprachoroidal device (Ayton et al., 2014). Currently, there are more than 350 individuals worldwide using Argus II devices (Ayton et al., 2020a). Although the production and implantation of the Argus II ended in 2019, there is ongoing research to develop other epiretinal devices (Ferlauto et al., 2018; Nano Retina, 2020).

The perceptual experience of clinically implanted Argus II patients has been variable (Erickson-Davis and Korzybska, 2021). In many patients a significant proportion of electrodes cannot elicit percepts within safe current density limits (Ahuja et al., 2013), and only limited pattern vision is generated by the device (Stronks and Dagnelie, 2014; da Cruz et al., 2016; Arevalo et al., 2021).

A variety of factors are likely responsible for the limited pattern vision found in Argus II devices (Caspi and Zivotofsky, 2015). These include the decoupling of retinotopic stimulation from eye-position (Caspi et al., 2017), the fact that the percepts produced by the electrodes are not well formed “pixels” (Nanduri et al., 2012; Luo et al., 2016; Beyeler et al., 2019), and an inability to resolve individual electrodes.

Two-point discrimination (the ability to determine whether one or two percepts are seen when a pair of electrodes are stimulated) is thought to be a particularly useful measure for characterizing the ability to resolve individual electrodes within an array (Ayton et al., 2020b). Unlike other spatial acuity tasks, such as grating acuity or square localization, two-point acuity is not susceptible to blurring by eye-movements. Thus, two-point discrimination is useful for characterizing losses in spatial resolution at a retinal level. An ability to resolve whether one or two electrodes have been stimulated is *necessary* but not *sufficient* for good visual performance with a prosthetic device; however, one previous study does suggests a correlation between two-point discrimination and grating spatial acuity in Argus II patients (Lauritzen et al., 2011).

**FIGURE 1:**
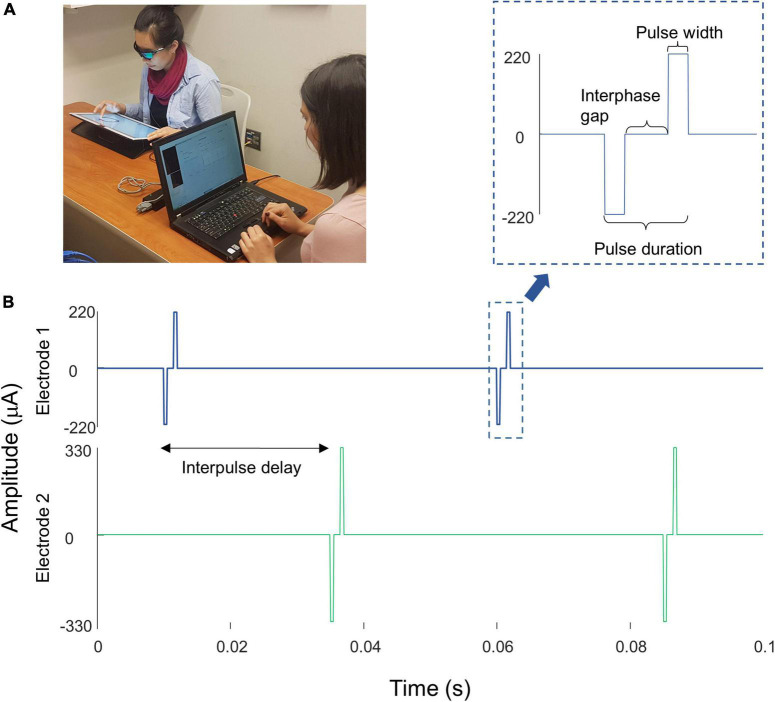
**(A)** Experimental setup. **(B)** Example pulse trains for an individual trial (not to scale) of the two-point discrimination paradigm. In all experiments we used square-wave, biphasic, cathodic-first pulse trains with a fixed pulse train duration.

Here we measured both current amplitude thresholds and two-point discrimination performance in three participants diagnosed with severe retinitis pigmentosa and chronically implanted with the Argus II epiretinal prosthesis (Table 1). Electrical stimulation was delivered directly to single or pre-selected pairs of electrodes (Figure 1). We measured single electrode thresholds using a yes-no procedure, and measured two-point discrimination thresholds by stimulating a pair of electrodes and asking participants both to report the number of phosphenes and draw the phosphene shape(s) on a tablet touch screen.

**TABLE 1:** Patient demographics.

Participant ID	Second sight participant ID	Implant	Eye	Age at testing	Date of implantation	Date of testing
S1	12-005	Argus II	Right Eye	83	2009	2019
S2	12-104	Argus II	Right Eye	61	2015	2019
S3	13-101	Argus II	Right Eye	74	2014	2019

All data reported in this paper were collected at Johns Hopkins University Wilmer Eye Institute.

Having measured current amplitude and two-point discrimination thresholds, we used a combination of regression analyses and simulations to examine the role of physical distance between electrodes, current amplitude, axonal stimulation, height of the electrode above the retinal surface (lift), and retinal damage, with the goal of examining how these various factors affect both sensitivity and two-point discrimination.

Our modeling section has three stages:

Stage I: ***Estimating electrode-electrode distance to and along axonal bundles***
  We began by using the *pulse2percept* model (Beyeler et al., 2019) to estimate the position of the array on the retinal surface, the distance between electrodes, and the distance to a shared axonal bundle for each pair of electrodes. These estimates were then used as predictive factors in our regression models (**Stage II**).
Stage II: ***Regression modeling: The effects of physical and axonal distance on two-point discrimination thresholds***
  Next, we fit nested linear logistic models to determine which factors—physical distance between electrodes, mean current amplitude of the two electrodes, and distance to axon (as estimated in **Stage I**), best predicted our psychophysical data.
Stage III: ***Current spread modeling: The effects of retinal damage and electrode lift on thresholds and two-point discrimination thresholds***
  Finally, we used a simple version of a “scoreboard” model to estimate the relative contributions of electrode lift from the retinal surface and retinal damage on both current amplitude thresholds and two-point discrimination performance. The scoreboard model assumes that each electrode generates a unitary, circular percept in the region of visual space that corresponds to the retinal position of that electrode, as would be predicted if electrical stimulation only elicited firing in ganglion cell bodies close to the electrode (Fine and Boynton, 2015).

## Psychophysics

### Methods

#### Participants

Our initial participant pool consisted of nine participants with Argus II retinal prostheses (Second Sight Medical Products, Inc.): six participants implanted and tested at the Retina Service at the University of Minnesota, and three tested at the Lions Vision Research and Rehabilitation Center at Johns Hopkins University; one of these patients was implanted at Wills Eye Hospital in Philadelphia, the other two at the Johns Hopkins Wilmer Eye Institute.

Unfortunately, five of the six University of Minnesota participants almost never reported seeing two percepts when stimulated with a pair of electrodes, and the one participant who did report seeing two percepts on a reasonable proportion of trials (7/49 in session 1; 35/90 in session 2) also reported seeing two percepts on 6/7 (no stimulation) catch trials. Consequently, we excluded the Minnesota participant data from further analysis.

Of the patients tested at Johns Hopkins, S1 was implanted as part of the Argus II Feasibility Study (clinicaltrials.gov trial NCT00407602), whereas S2 and S3 were implanted after Argus II became commercially available in 2013, see Table 1. The data described in this paper (Johns Hopkins participants) were collected in two sessions, with each session taking roughly 3 h, including frequent breaks.

Data were collected at the Retina Service at the University of Minnesota and the Lions Vision Research and Rehabilitation Center at Johns Hopkins University, and were provided to UW researchers in a de-identified format. The study was approved by IRBs of the University of Washington and Johns Hopkins University.

#### Stimuli

The Argus II retinal prosthesis consists of an epiretinal electrode array implanted in the macular region of the retina, an ASIC chip, RF transmitter and receiver coil, as well as glasses containing a mini camera (not used in our experiment) and a video processing unit (VPU), see Ahuja et al. (2011), for more detail. The signal from the VPU is received by the internal receiver coil, and the ASIC chip generates the electrical pulses that are then sent to the electrode array, a grid of 6 × 10 platinum disk electrodes in a rectangular grid arrangement with 225 μm diameter and 575 μm center-to-center separation.

We stimulated electrodes directly by connecting the VPU of each participant’s device to a psychophysical testing computer provided by Second Sight Medical Products, Inc., see Figure 1A. Electrode stimulation was controlled by in-house software programmed in MATLAB by Second Sight Medical Products, Inc. (Mathworks, MATLAB Version: 7.1, R14SP3), that sent current waveforms directly to the electrodes (by-passing the camera). Stimuli consisted of biphasic, cathodic-first, charge-balanced, square-wave pulse trains with frequency, interphase gap, interpulse delay (the offset between pulses on different electrodes) and pulse train duration parameters as shown in Figure 1B and Table 2. Stimulation current amplitudes were kept below a charge density limit of 1 mC/cm^2^/phase.

**TABLE 2:** Stimulation protocol and parameters for all experiments.

	Patient ID	Experiment	Frequency (Hz)	Interphase gap (ms)	Interpulse delay (ms)	Duration (ms)
1^st^ Session	S1	Perceptual Threshold	20	0	N/A	250
	S2	Perceptual Threshold	6	1	N/A	250
	S3	Perceptual Threshold	6	1	N/A	250
	S1	Two-point Discrimination	20	0	25	250
	S2	Two-point Discrimination	20	0	25	250
	S2	Two-point Discrimination	6	0	83	500
	S3	Two-point Discrimination	6	1	83	500
	S3	Two-point Discrimination	6	1	83	500
2^st^ Session	S1	Perceptual Threshold	20	0	N/A	250
	S2	Perceptual Threshold	6	1	N/A	500
	S3	Perceptual Threshold	6	1	N/A	500
	S1	Two-point Discrimination	6	1	83	500
	S2	Two-point Discrimination	6	1	83	500
	S3	Two-point Discrimination	6	1	83	500

#### Identifying electrodes with lower perceptual thresholds

A proprietary fast threshold estimation procedure, SwiftPA (Second Sight Medical Products Inc, 2013) was used to determine which electrodes had electrical thresholds below the safety limit. Stimulation consisted of 0.46 ms, cathodic-first pulse trains of 1 s duration. Starting from the top left electrode, a yes-no procedure was used to determine whether stimulation produced a detectable phosphene. If participants failed to detect a phosphene the amplitude of the electrical stimulation was increased. If participants reported a phosphene the amplitude was held constant. After 3 consecutive correct detections, testing moved to the next electrode. We limited further testing to a subset of the electrodes which produced 3 consecutive correct detections at a current amplitude below the safety limit (10 electrodes in S1, 7 electrodes in S2, and 10 electrodes in S3). These electrodes were selected to have low thresholds, and to be spread as widely apart as possible on the array.

#### Current amplitude detection threshold measurements

We then used proprietary software (Argus II-Hybrid Threshold) provided by Second Medical Products Inc. to carry out an adaptive, single interval yes-no procedure to measure detection thresholds (50% detection performance) within electrodes pre-selected by the SwiftPA procedure, methodological details are explained more fully in Ahuja et al. (2013).

To avoid adaptation effects (Horsager et al., 2009; Pérez Fornos et al., 2012) we interleaved threshold measurements across electrodes within each run. Up to six electrodes were tested within a single run. Each trial started with an audio prompt. Then one of the six selected electrodes (selected pseudorandomly) was stimulated at either 20 or 6 Hz, with a pulse train duration of either 250 or 500 ms (depending on what the participants used in their daily life, see Table 2), square-wave pulse width of 0.46 ms, and interphase gap of 0 or 1 ms. The amplitude of stimulation was adapted through a staircase procedure. The participant was asked to report whether or not they had seen a phosphene on that trial using a game controller and feedback was given on each trial. Each run consisted of a maximum of 60 trials per electrode (5 blocks of 12 trials), for a maximum of 360 trials, and 4 catch trials per block (Second Sight Medical Products Inc, 2013). Each run was followed by a brief rest, which ended based on participant feedback.

Perceptual thresholds for detection at a given electrode were calculated by pooling data across all trials. The probability of reporting a percept as a function of stimulus intensity was fit with a psychometric function using maximum likelihood estimation, and the current amplitude detection threshold was defined as the stimulus amplitude at which the participant reported a percept 50% of the time (Watson and Robson, 1981; Wichmann and Hill, 2001; Ahuja et al., 2013).

#### Two-point discrimination measurements

For each participant, we selected electrodes with the lowest detection thresholds and paired them in all possible combinations. Stimulation was carried out at an amplitude twice the detection threshold, or at a maximum of 660 μA (the charge density limit of 1 mC/cm^2^/phase for a 0.46 ms pulse). On each trial, we asked “how many shapes did you see” and asked them to draw the phosphene shape(s) on a tablet touch screen.

Parameters used for each participant are shown in Table 2. The pulse width was always 0.46 ms, with an interphase gap of 1 ms for S2 and 3, and no interphase gap for S1, based on the stimulation parameters each individual was accustomed to through daily use. Stimulation was interleaved, with either a 25 ms (20 Hz) or 83 ms (6 Hz) interpulse delay between the beginning of each pulse on one electrode and the beginning of the corresponding pulse on the second electrode, Figure 1B.

In each experimental run, every possible pair of electrodes was tested 3 times. On each trial, participants verbally reported the number of shapes they were seeing, gave a qualitative description (e.g., “the one on the bottom is smaller,” “left one is twice as big as right one—they are side by side”) and traced the perceived phosphene shape(s) on a tablet (drawing data not reported here). Although the “correct” answer was always two percepts, participants were never given feedback as to how many electrodes had been stimulated. Importantly, the number of shapes drawn by the participant was almost always consistent with the number of shapes they verbally reported, suggesting that they were not reporting whether they saw one or two shapes on the basis of the overall brightness or size of the percept.

We included ∼25% of catch trials, randomly interspersed, in which neither of the electrodes was stimulated. We deliberately used no stimulation as compared to single electrode stimulation during catch trials, because we were concerned that differences in brightness or size might allow participants to differentiate between single and paired stimulation in the absence of genuine pattern vision (see section “Discussion”).

The order of the trials was pseudorandomized. We asked participants to avoid head and eye movements to maximize stability of the perceived phosphene locations, but to maximize participant comfort we did not use a chin rest.

### Results

#### Current amplitude detection thresholds

**FIGURE 2:**
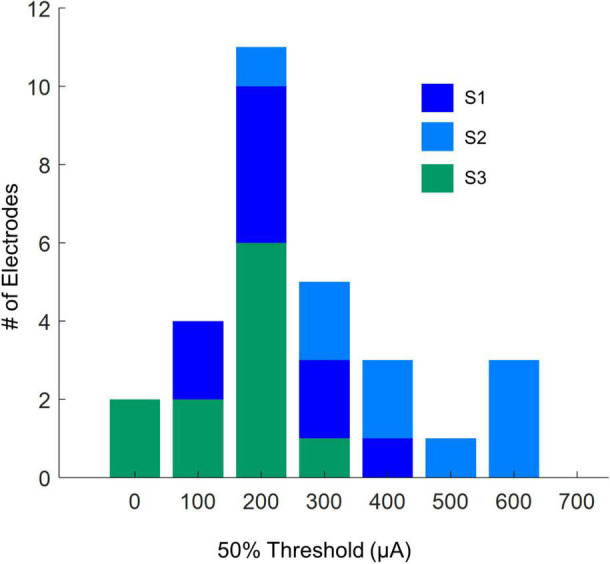
Histogram of current amplitude detection thresholds (50% detection performance).

Table 3 shows 50% current amplitude detection thresholds and self-reported daily usage for all three participants, and Figure 2 shows a histogram of current amplitude thresholds for all participants.

**TABLE 3:** Current amplitude detection thresholds (50% detection performance) and reports of daily usage.

	Median threshold (μA)	Interquartile range (μA)	Minimum threshold (μA)	Maximum threshold (μA)	Daily use
S1	274	218–331	153	484	3–4 days a week, 3–5 h a day, for an average of 20 h a week
S2	476	355–621	217	645	Approximately once a month
S3	210	177–280	89	323	Used the device almost every day outdoors, for a limited amount of time, averaging about 2 h a day.

#### Two-point discrimination thresholds

In the paired electrode stimulation experiment, we asked the question “*How many shapes did you see?”* Participants could potentially report any value, and they were then asked to draw what they saw. Table 4 shows the reported number of percepts and the probabilities of each verbal response.

**TABLE 4:** Reported number of percepts and their frequency and probability in the two-point discrimination experiment.

	Reported number of percepts	Frequency	Probability P(“X”|2)
S1	“0”	0	0.00
	“1”	66	0.31
	“2”	114	0.69
	“3”	0	0.00
S2	“0”	2	0.04
	“1”	29	0.54
	“2”	20	0.37
	“3”	3	0.06
S3	“0”	0	0.00
	“1”	38	0.36
	“2”	67	0.64
	“3”	0	0.00

Trials where participants did not report seeing a percept were discarded (0–4%). The few trials where participants reported 3 percepts (0–6%) or more percepts were collapsed with trials where participants reported 2 percepts for the remainder of the analyses.

Given that we always used paired electrode stimulation, we were concerned that participants might shift their criterion for reporting two percepts over the course of the experiment. However, there was little evidence that the probability of participants reporting two (or more) percepts changed substantially either within or across sessions (although it should be noted that electrode pairs varied between sessions, Table 5, which may have masked some experience driven effects). For S3 there was a significant increase in the probability of reporting two percepts between the first and second ½ of trials in session 2. The reason for this is not clear, but might possibly be due to an improved ability to recognize two percepts with experience. Since there was little effect of time on our two-point discrimination data we did not use time as a factor in our further analyses. The number of shapes participants drew consistently matched their verbal report, throughout every session.

**TABLE 5:** Probability of reporting two percepts, within and across sessions.

Participant	Session	1st half of trials in session	2nd half of trials in session	Unique electrodes tested
S1	Session 1	0.73 [0.48,0.89]	0.93 [0.7, 0.99]	A4, A8, D1, E10, F2
	Session 2	0.68 [0.57, 0.77]	0.50 [0.4, 0.62]	A2, A4, A8, B3, B6, D1, D8, E10, E3, F2, F7
S2	Session 1	0.33 [0.12, 0.65]	0.56 [0.27, 0.81]	B6, B9, F7, F9
	Session 2	0.39 [0.2, 0.61]	0.44 [0.25, 0.66]	A10, B10, B5, B6, B9, F7, F9
S3	Session 1	0.71 [0.25, 0.66]	0.59 [0.41, 0.74]	A8, B10, B4, C6, C8, C9, D6, E9, F10
	Session 2	0.35 [0.19, 0.55]	0.91 [0.72, 0.97]	A6, A8, B10, B9, D6, F10

Values in the bracket are 95% confidence intervals calculated by Wilson method.

The probability of reporting 1 or 2 (or more) percepts during catch trials (with no stimulation) also remained reliably low throughout the experiment (S1:0/15 trials, S2:2/12 trials, S3: 0/16 trials).

#### Optical coherence tomography data

**FIGURE 3:**
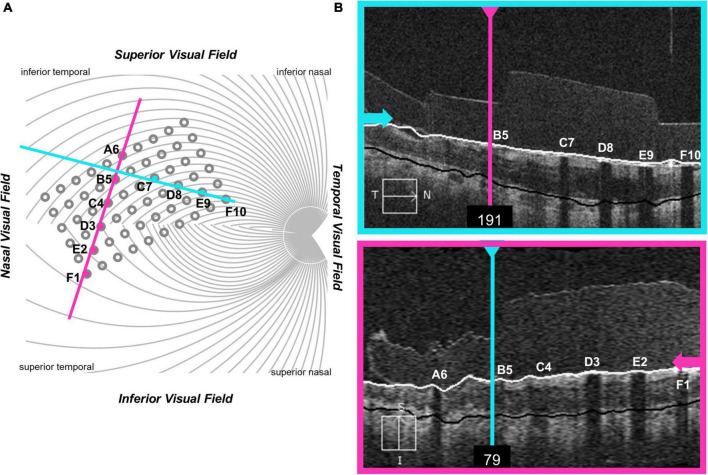
OCT data from participant S1. **(A)** Schematic showing the estimated location of OCT b-scans overlaid on the array (alignment was carried out using the registered OCT fundus image). The array schematic is flipped along the y-axis to reflect visual space coordinates, such that the top of the schematic represents the superior visual field and the inferior retina. **(B)** The metal electrodes block light from the scanning light source, casting shadows on the retinal image.

Unfortunately, it was impossible to collect useable optical coherence tomography (OCT) images for the region of the retina including the array in S2 and S3. OCT data from S1, 2 years after implantation (2011), are shown in Figure 3. In this patient the array appears to be flush to the retinal surface, but there is some thickening (evidence of potential damage) of the retina underneath the array.

#### Relationship with device use

In a separate questionnaire, participants were asked how often they used their device. Participants varied widely in device use, see Table 3. S1, who had a median amplitude threshold of 274 μA and saw 2 percepts 69% of the time with paired stimulation, used the device most consistently, reporting using the device 3–4 days a week, 3–5 h a day, for an average of 20 h a week. S3, who had a median amplitude threshold of 210 μA and saw 2 percepts 64% of the time with paired stimulation, used the device almost every day when outdoors, but for a limited amount of time, averaging about 2 h a day. S2, who had a median amplitude threshold of 476 μA and saw 2 percepts only 37% of the time with paired stimulation, reported using the device once a month.

## Modeling

Pulse2percept analyses and current amplitude threshold estimates were carried out in Python using pulse2percept (Beyeler et al., 2017) and in-house code. The remaining analyses were carried out in MATLAB (Mathworks, MATLAB Version: 9.10.0, R2021a) using in-house code. All in-house code can be found at https://github.com/VisCog/Argus_current_spread.

### Stage I: Estimating electrode-electrode distance to and along axonal bundles

As an initial step we estimated distance to and along axonal bundles for each pair of electrodes.

Both electrophysiological (Fried et al., 2009) and psychophysical data (Beyeler et al., 2019) suggest that axonal stimulation may contribute significantly to the poor resolution of retinal prostheses. Axonal stimulation is a particular concern for epiretinal prostheses, such as the Argus II which are placed on the nerve fiber layer, adjacent to the axon fiber bundles of retinal ganglion cells. Depending on stimulus conditions, participants implanted with the Argus II describe the phosphenes generated by single electrodes as elongated, due to activation of passing axon fibers, resulting in perceptual distortions (individual electrodes producing “streaks” instead of punctate spots) that vary in their length and orientation across the retinal surface in a way that can be predicted based on the known axon fiber trajectories (Nanduri et al., 2012; Beyeler et al., 2019).

It is not yet entirely clear how sensitivity to electrical stimulation falls off as a function of distance from the initial segment (Fried et al., 2009), with psychophysically estimated decay constants ranging widely from 500–1,420 μm (Beyeler et al., 2019). Nonetheless, if axonal stimulation plays a significant role in reducing resolution, then distance, both to and along a shared axon bundle should predict how many distinct percepts are seen when two electrodes are stimulated.

#### Methods

To provide a measure of the distance to and along axon bundles we used an existing computational model developed by Beyeler et al. (2019). This model begins by using ophthalmic fundus photographs in which an eye care provider marked the optic nerve, fovea, and the center of the implant on the fundus, using photos taken pre- and post-surgery. These landmarks were then used to estimate the array center with respect to the fovea, the array rotation with respect to the horizontal raphe, and the retinal distance between the fovea and the optic nerve head for each participant. In the human retina, the extended raphe is typically located 15° ± 2° inferiorly to a horizontal line at the latitude of the fovea through the center of the optic disk. We approximated this by fitting a parabola centered on the optic nerve and approximating the horizontal raphe as parallel to the axis of symmetry on the abscissa (Jansonius and Schiefer, 2020).

**FIGURE 4:**
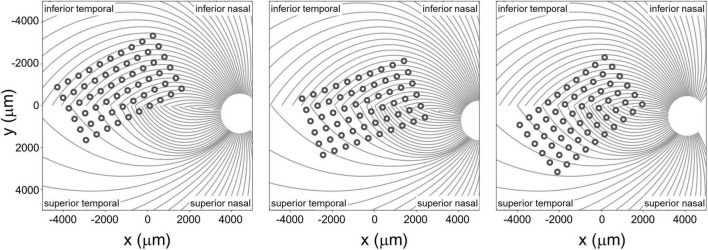
Estimated position of the electrode array on the retinal surface for all three participants (see Beyeler et al., 2019 for estimation methods) overlaid on estimates of the axon fiber pathways for that participant. Note that all panels are in visual space coordinates, with the upper visual field at the top of the figure.

The spatial layout of axonal pathways was calculated using *pulse2percept* software (Beyeler et al., 2017), that simulates pathways using a model (Jansonius et al., 2009) that assumes that the trajectories of the optic nerve fibers can be described in a modified polar coordinate system (*r*,ϕ) with its origin located in the center of the optic disk. Each nerve fiber is modeled as a spiral defined by the angular position of the trajectory at its starting point at a circle around the center of the optic disk, with a second parameter describing the curvature of the spiral, see Figure 4.

Given that the size of the Argus II electrodes is large compared to the density of the underlying axon pathways, it was assumed that an electrode always sits on top of a ganglion axon fiber bundle. We simulated 400 axonal bundles, which provided sufficient resolution to ensure that there was an axonal bundle underneath every electrode.

**FIGURE 5:**
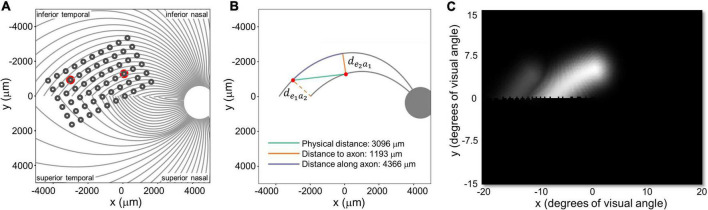
**(A)** Estimated position of the electrode array on the retinal surface for S1 (replotted from Figure 4A). **(B)** Examples of distance to and along axon fibers, *d*_*e*_1_*a*_2__ and *d*_*e*_2_*a*_1__ refer to the shortest distance from an electrode to the axonal bundle closest to fellow electrode. **(C)** Predicted percept for the two electrodes shown in panels **(A,B)**. Note that all panels are in visual space coordinates, with the upper visual field at the top of the figure.

We defined three inter-electrode distance values, Figure 5:

1. ***Physical distance*** was defined as the Euclidean center-to-center distance between two electrodes on the retinal surface.
2. ***Distance to axon*** (*d*_*axon*_12__) was defined as the minimum distance between two electrodes (e_1_, e_2_) and the axons closest to them. This was calculated by:
  a. Selecting the axonal bundles a_1_ and a_2_ that fell beneath each of the two electrodes.
  b. Determining the closest Euclidean distance from the center of each electrode to the fellow axon bundle: *d*_*e*_1_*a*_2__ = min [*d*(*e*_1_, *a*_2_)] and *d*_*e*_2_*a*_1__ = min [*d*(*e*_2_, *a*_1_)]
  c. Choosing the minimum distance of the pair, *d*_*axon*_12__ = min[*d*_*e*_1_*a*_2__, *d*_*e*_2_*a*_1__].
  (An alternative would have been to calculate the distance to the axon bundle midway between the two electrodes, but this would have essentially resulted in the same values, halved).

3. ***Distance along axon*** was defined as the distance between an electrode and the point on its axon that is closest to the axon of the other electrode.

#### Results

These three measures of distance on the retina were strongly correlated with each other. The Pearson correlation coefficient between physical distance and distance to axon was r(337) = 0.58, *p* < 0.0001, between physical distance and distance along axon was r(337) = 0.84, *p* < 0.0001, and between distance to axon and distance along axon was r(337) = 0.28, *p* < 0.0001.

Intuitively, the reason for this is that (except when electrodes are on the opposite side of the Raphe) the shortest distance to the axon (orange line in Figure 5B) tended to fall along a line that was close to orthogonal to the distance along the axon (purple line), since axonal bundle curvature (purple line) tended to be relatively small. As a result, these three distances form the edges of an approximate right triangle, with the hypotenuse as the Euclidean distance between two electrodes (green line) and distance to and along the axon (with a slight curvature) forming the other two sides. Because these three co-varying distance variables essentially contain 2 degrees of freedom, we only included physical distance and distance to axon as predictive factors in our modeling.

### Stage II: Regression modeling: The effects of spatial and axonal distance on two-point discrimination thresholds

Next, we fit nested linear logistic models to determine which factors—physical distance between electrodes, mean amplitude of the currents of both electrodes, and distance to axon (as estimated in modeling stage **I**), best predicted our psychophysical data.

#### Methods

The probability of participants reporting 2 (or more) shapes when 2 electrodes were stimulated, P(‘2’| 2), was modeled using logistic regression. We used a maximum likelihood chi-squared test to determine whether adding parameters improved model fits. Across all analyses that included current amplitude as a factor, subject identity had little additional predictive value and so it was not included as a factor.

Regression was done both using a two-factor model with inter-electrode physical distance and mean stimulation amplitude of the two electrodes as predictors, and with a three factor model that included distance to axon across the pair of electrodes as a third predictor.

#### Results – two factor model

We began with a two factor model that included (1) physical distance and (2) the mean stimulation amplitude of the two electrodes as predictors.

A maximum likelihood chi-squared test shows that both factors statistically improved the fit to the data, Table 6.

**TABLE 6:** Logistic regression model parameters and statistical significance.

		Estimate	95% CI		χ^2^(1)	Pr(>χ^2^)
			Lower	Upper		
2-factor model	Amplitude	–0.003014	–0.005144	–0.000883	7.87	0.005022
	Physical Distance	0.000829	0.000575	0.001084	50.64	< 0.0001
3-factor model	Amplitude	–0.003	–0.005145	–0.000840	7.61	0.005802
	Physical Distance	0.000602	0.000309	0.000895	18.40	< 0.0001
	Distance to Axon	0.000503	–0.000876	–0.000129	7.20	0.007279

The intercepts are not included in the table, but are included in Equations 1, 2.

The best-fitting two-factor model predicts the probability of seeing two percepts as:


(1) P("2"|2)=exp(-0.0599-0.00314(MeanAmplitude)+

0.000829(PhysicalDistance))

**FIGURE 6:**
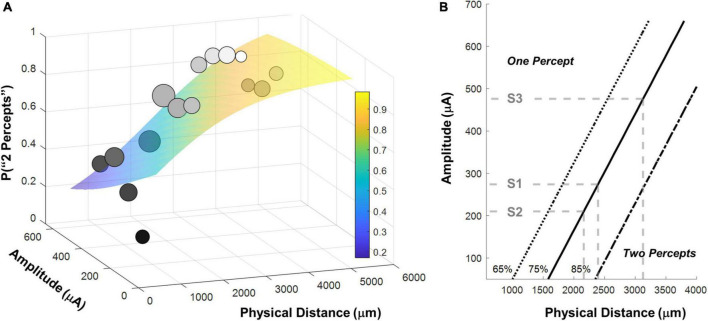
**(A)** Surface predicting the probability of reporting 2 percepts using logistic regression with mean detection threshold amplitude and physical distance as fixed factors. Individual data points are generated by binning the data (collapsed across participants) and calculating the probability of seeing two percepts in each bin. The gray-scale shade of the data point represents the probability of seeing two percepts, the size of the data point represents the number of observations in that bin. Surfaces were fit to the original trial-by-trial un-binned data. **(B)** 65%, 75% and 85% two-point discrimination iso-performance curves as a function of amplitude and physical distance. Gray dashed lines show predicted two-point discrimination thresholds for electrodes at the median current amplitude threshold values for S1-S3.

Figure 6A shows the binned participant performance values and the surface predicting the probability of reporting 2 percepts based on the logistic regression model. Figure 6B shows predicted 65%, 75% and 85% two-point discrimination iso-performance curves based on the surface of Figure 6A.

**FIGURE 7:**
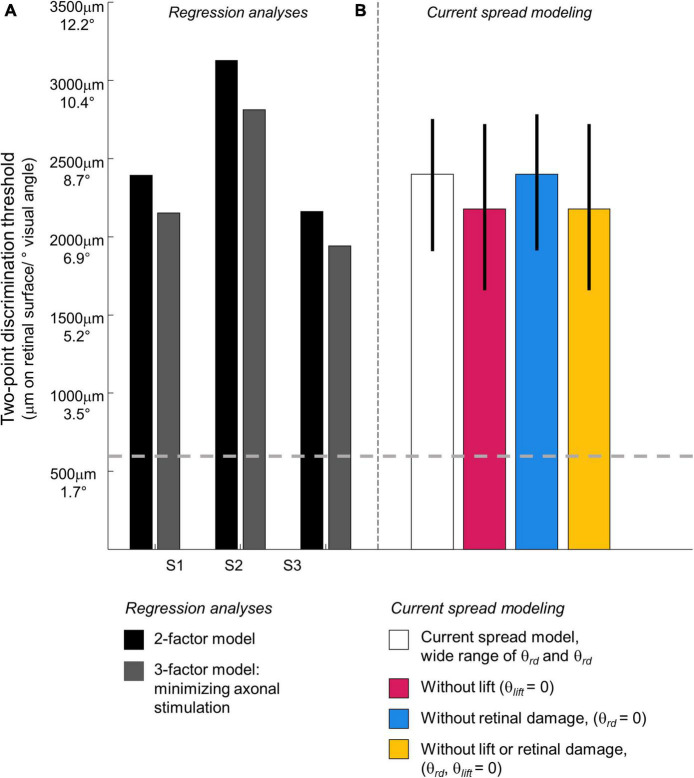
Predicted two-point discrimination thresholds. The gray dashed line shows the resolution limit (2°) that would be obtained if individuals saw two-points when neighboring electrodes were stimulated (Stronks and Dagnelie, 2014). **(A)** *Stage II. Regression analyses.* Black bars: The predicted 75% two-point discrimination thresholds with current amplitudes set to each participant’s median current amplitude detection threshold; Gray bars: The predicted 75% two-point detection thresholds if axonal stimulation is minimized. **(B)** *Stage III. Current spread modeling.* Empty bar: Predicted 75% two-point discrimination thresholds across the full range of simulation parameterizations that could predict the iso-performance contour predicted by regression analyses; Blue bar: Predicted 75% two-point discrimination thresholds with no retinal damage (θ*_*rd*_* = 0); Pink bar: Predicted 75% two-point discrimination thresholds with the electrode flush to the retinal surface (θ*_*list*_* = 0); Yellow bar: Predicted 75% two-point discrimination thresholds with no retinal damage and the electrode flush to the retinal surface (θ*_*rd*_*, θ*_*list*_* = 0). Error bars represent the 5–95% confidence range of simulation outcomes.

As expected, the probability of seeing two percepts increased as a function of physical distance and decreased as a function of mean amplitude. We used this two-factor logistic regression model fit, whose surface is shown in Figure 6A, to define the two-point discrimination threshold as the inter-electrode distance for which participants should report two percepts on 75% of trials at each participant’s median current amplitude detection threshold (S1 = 2,394 μm/8.3°, S2 = 3,127 μm/10.9°, S3 = 2,161 μm/7.5°; reported in microns on the retina and degrees of visual angle, assuming a conversion of 288 μm = 1° (Drasdo and Fowler, 1974), shown with black bars in Figure 7A. For comparison, the approximate size of the Argus II prosthetic array was 3,675 × 5,975 μm/12.8 × 21°, with a distance between neighboring electrodes of 575 μm/2°. Thus, a spacing of about four electrodes is needed to report two percepts on 75% of trials.

#### Results – three factor model

Distance to axon also had significant predictive value, as shown in Table 5. Within this 3-factor model, the ability to predict whether one or two percepts were reported was best modeled as:


(2) P("2"|2)=exp(-0.1839-0.0030(MeanAmplitude)+

0.000602(Distance)+0.000502581(Distancetoaxon))

As noted above, physical distance and distance to axon were strongly correlated. Correlations between independent variables do not reduce the predictive power of a model but it becomes difficult to disentangle the separate effects of each explanatory variable on the explained variable (Kutner et al., 2004). Thus, the beta weights of regression Equation 2 should be interpreted with caution.

**FIGURE 8:**
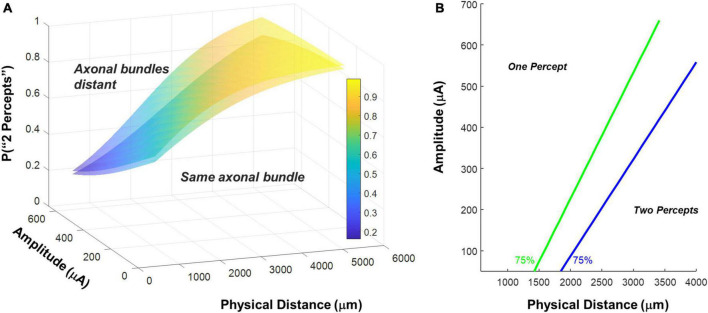
**(A)** Surfaces based on fitting the probability of reporting 2 percepts using logistic regression with amplitude and physical distance as fixed factors. Having fixed the weights for amplitude and physical distance, we included distance to axon as a factor. The lower surface represents predictions for distance to axon = 0 (the two electrodes fall on the same axonal bundle), the upper surface represents predictions for distance to axon = physical distance (minimizing axonal stimulation). **(B)** 75% two-point discrimination iso-performance curves as a function of amplitude and physical distance for distance to axon = 0 (the two electrodes fall on the same axonal bundle), and distance to axon = physical distance (minimizing axonal stimulation).

Therefore, to estimate the size of the effect of axonal stimulation on two-point discrimination thresholds we began with the 2-factor regression model described in Equation 1 and Figure 6, fixed the best-fitting factor weights for these two factors, then added distance to axon as an additional factor. This allowed us to calculate the probability of reporting two percepts when the distance to axon was zero (i.e., the two electrodes fell on the same axon bundle, shown in the lower surface of Figure 8A) vs. when the distance to axon was equal to the physical distance (i.e., axonal stimulation was minimized, upper surface of Figure 8A).

Figure 8 shows 75% iso-performance contours for these upper and lower surfaces. As expected, the effects of axonal stimulation are smaller when the physical distance between the two electrodes is small. According to the model, if axonal stimulation were minimized, the physical distance between the electrodes that would result in a 75% two-point discrimination threshold for each participant’s median current amplitude threshold would be S1 = 2,151 μm/7.5°, S2 = 2,812 μm/9.8°, S3 = 1,942 μm/6.7°, respectively, shown by the dark gray bars in Figure 7. This corresponds to a reduction in the two-point distance threshold of ∼0.8° for S1 and S3, and ∼1.1° for S2.

### Stage III: Current spread modeling: The effects of retinal damage and electrode lift on thresholds and two-point discrimination thresholds

Having estimated the effect of axonal stimulation on two-point discrimination performance, we simulated a simplified “scoreboard” model to identify how retinal damage and lift might influence amplitude and two-point discrimination thresholds. According to the scoreboard model, the main determinant of whether one or two percepts are seen will be the overlap of the current fields generated by the electrodes on the retinal surface. This overlap is affected by the physical distance between the electrodes along the retinal plane, “lift” of the electrodes from the retinal surface, and the current amplitude on the electrodes.

As lift increases, so does the current amplitude required to elicit a percept. A larger current amplitude at a greater distance from the retina results in a broad current spread on the retinal surface. Extensive psychophysical work with early participants implanted with the Argus I and II devices has shown that, for individual electrodes, it is possible to predict the size, threshold and brightness of suprathreshold phosphenes as a function of frequency and amplitude, with reasonable accuracy, once the height of the electrode off the retinal surface is included as a factor (de Balthasar et al., 2008; Horsager et al., 2009; Nanduri et al., 2012; Ahuja et al., 2013).

Various types of retinal damage may also increase the current needed to generate a percept and thereby affect amplitude thresholds and possibly two-point discrimination. Possible causes of retinal damage include severe disease-related degeneration, damage to the retina as a result of surgical implantation, or damage caused by the presence of the array. A variety of studies have found evidence suggestive of retinal damage in retinal prosthesis patients (Gregori et al., 2018; Lin et al., 2019; Rizzo et al., 2019; Patelli et al., 2020). This damage seems to have a variety of causes including inflammation, “boggy” (*sic*) thickening, schisis and fibrosis, intraretinal fluid (IRF) cysts, as well as a “snowplow” effect of the electrode array pressing against the retina and causing adjacent thickening (Gregori et al., 2018; Patelli et al., 2020). While many of these conditions are common in late stage RP patients, they seem to be exacerbated in the implanted eye (Lin et al., 2019). In addition, over time many patients also develop membranes (both adherent to and separated from the retina) between the retina and the array. For example, Patelli et al. (2020) observed in one patient the formation of retinal fibrosis and schisis within 2 years of implantation which resulted in higher thresholds in 34 out of 60 electrodes. After the removal of retinal fibrosis, 20 out of 60 electrodes were reactivated; suggesting this fibrosis was responsible for reducing electrode sensitivity.

#### Methods

We simulated current spread as a function of 3D distance from the edge of the electrode as follows:


(3) $$I_{\mathrm{xyz}}=\frac{I_{0}}{1+{\left(\mathrm{kr}\right)}^{a}}$$

Where *I_0_* is the stimulating current and *r* is the 3D distance from the edge of the electrode (Ahuja et al., 2008). Parameters *k* and *a* describe current spread. The range of *k* and *a* values were chosen to approximate previous psychophysical data describing threshold as a function of lift (de Balthasar et al., 2008; Ahuja et al., 2013), and be consistent with more elaborate neurophysiological models (Esler et al., 2018). The parameter *a* varied between 1–3, and *k* varied between 6–20, providing a parameterization of current spread that widely spanned the neurophysiologically plausible range. For both *a* and *k*, larger values represent higher amounts of tissue electrical resistance, so current amplitudes drop more quickly as a function of *r*.

**FIGURE 9:**
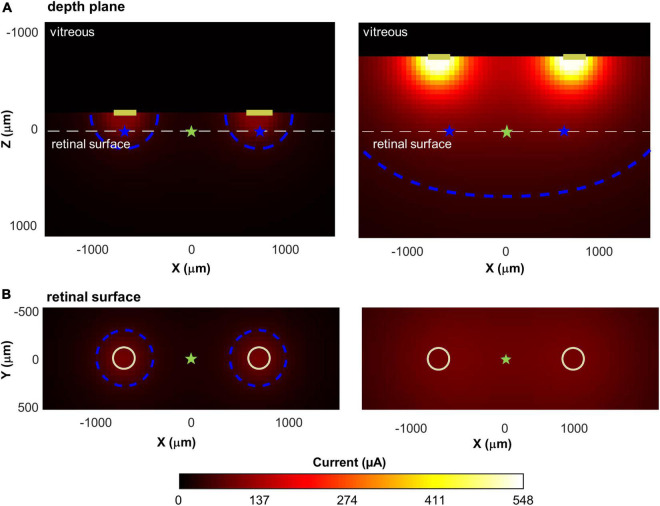
Illustration of current spread for two pairs of disk electrodes, separated by 1,400 μm, with different lifts from the retinal surface. Top: Cross-sectional view through the retina; Bottom: View of the retina from above. Current amplitude at the electrode was fixed to produce a maximum of 100 μA of current on the retina. **(A)** Lifted 150 microns above the retinal surface. **(B)** Lifted 750 microns above the surface. The white dashed line in the upper panels shows the location of the retinal surface. Blue contour lines in each panel represent 50 μA; cyan star: the intermediate point between the electrodes; blue star: the point of maximum current on the retinal surface.

Figure 9 shows two example simulations for a pair of electrodes, separated by *d* = 1,400 μm, *a* = 1.5, *k* = 15, lifted by 150 μm and 750 μm above the retinal surface. The higher an electrode is lifted off the retinal surface, the greater the electrode current required to produce an electric field gradient sufficient to elicit spikes in the axons passing through the retinal surface. The bottom panels represent a top view, showing current at the retinal plane. Both simulations of Figure 9 have a maximum current value of 100 μA at the retinal surface, however, the region of high current is much broader for the electrode pair that are lifted 750 μm above the retinal surface.

Thus, for electrodes that are lifted off the surface, the increased overlap between the current fields is likely to reduce the ability to differentiate two distinct phosphenes. We represented this overlap by calculating the decrease in current amplitude at the point intermediate between the electrodes (I_mid_, cyan stars) compared to the point of maximum current (I_max_, blue stars), on the retinal surface. This “dip” in current was calculated as:


(4) $$\mathrm{dip}=100\left(\frac{I_{\max}-I_{\mathrm{mid}}}{I_{\max}}\right)$$

In Figure 9, when electrodes are lifted 150 μm above the retinal surface (Panel A), I_max_ = 100 μA, I_mid_ = 26 μA, *dip* = 74%, whereas when electrodes are lifted 750 μm above the retinal surface (Panel B), I_max_ = 100 μA, I_mid_ = 93 μA, *dip* = 7%.

We assumed that the measured threshold current for seeing a percept at an electrode (*I*_0_) could be described as a multiplicative combination of three factors:


(5) $$I_{0}=\theta_{\mathrm{lift}}\cdot \theta_{\mathrm{rd}}\cdot \theta_{\mathrm{baseline}}$$

θ*_*baseline*_* is the current required to elicit enough spikes to reach psychophysical threshold for an electrode flush to the retinal surface in an RP patient whose retina is undamaged. We fixed θ*_*baseline*_* = 50 μA, based on the maximum sensitivity observed in previous psychophysical data (de Balthasar et al., 2008; Horsager et al., 2009; Ahuja et al., 2013).

θ*_*lift*_* represents a multiplicative increase in electrode current required as a result of the electrode being lifted above the retinal surface. The value of θ*_*lift*_* monotonically increases as a function of lift, with a non-linear curve that depends on *a* and *k*, as described by Equation 3.

θ*_*rd*_* represents an additional multiplicative increase in the current amplitude required to reach threshold, which we propose is likely due to various types of retinal damage.

We simulated a wide range of *k, a*, θ*_*rd*_*, and θ*_*lift*_* (corresponding to lifts of 0–1,000 μm) for single electrodes. For each combination of parameters, we used least squares function minimization to find the electrode current, I_0_, required to reach threshold for that parameterization.

We then simulated pairs of electrodes across a wide range of physical distances (*d* = 250–8,000 μm). Stimulation amplitude was fixed at twice threshold for that parameterization (or 660 μm, whichever was smallest). For each parameterization we calculated *dip*. A final parameter, *dip criterion*, is the dip value that results in a 75% two-point discrimination performance. We assumed that a 75% probability of seeing two percepts required a dip criterion > 20%.

#### Results

**FIGURE 10:**
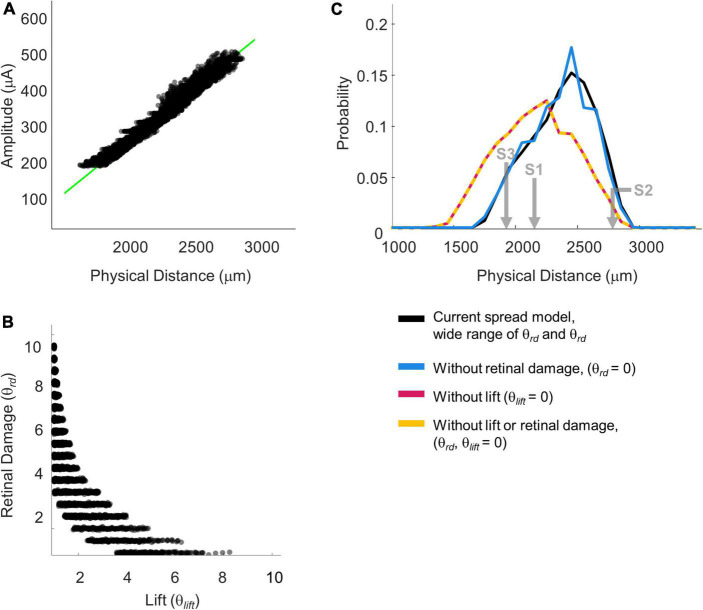
Simulation parameterizations that match participant performance. **(A)** The green line representing 75% iso-performance without axonal stimulation is replotted from Figure 6. Multiple overlapping black lines show simulated iso-dip contours sub-selected from parameterizations that matched the 75% two-point discrimination iso-performance curve. **(B)** Scatter plot of θ_*rd*_ vs. θ_*lift*_ for parameterizations which resulted in iso-dip contours [the black lines in panel **(A)**] that closely resembled the predicted 75% iso-performance contour with minimal axonal stimulation [the green line in panel **(A)**]. **(C)** Probability distributions of predicted two-point discrimination thresholds.

Across each simulated value of *a, k*, θ*_*rd*_*, θ*_*lift*_*, and *dip criterion* we calculated both the predicted detection threshold amplitude and the physical distance that produced *dip* = *dip criterion*. From these simulations we created iso-dip contours as a function of physical distance and I_0_. We sub-selected those simulated iso-dip contours that were reasonably close (mean squared error < 20 μA) to the predicted 75% iso-performance contour for minimal axonal stimulation and whose parameterizations resulted in single electrode thresholds between 177 and 660 μA. These successful parametrizations are shown in Figure 10A, with the green line representing the estimated 75% iso-performance curve for minimal axonal stimulation from the regression analyses described earlier, replotted from Figure 8B.

Current amplitude thresholds in our patients were consistently higher (by a factor of 4–12x) than 50 μA (see Table 3), suggesting that θ*_*rd*_* and/or θ*_*lift*_*, play an important role in determining threshold. This is confirmed by the scatter plots of θ*_*rd*_* vs. θ*_*lift*_* for successful simulations, Figure 10B, where there is an absence of scatter points with both low θ*_*rd*_* and θ*_*lift*_*. Although the broad range of plausible outcomes generated by our simulations makes it difficult to definitively attribute the degree to which elevated thresholds and an inability to resolve individual electrodes can be attributed to retinal damage vs. lift; our simulations suggest that both damage and/or lift may play a role.

Next, using the parametrizations that successfully predicted two-point discrimination performance, we examined how retinal damage (θ_*rd*_) and lift from the retinal surface (θ_*lift*_) affected spatial two-point discrimination thresholds. We calculated predicted two-point discrimination thresholds across all values of a, k, θ_*rd*_, θ_*lift*_, and *dip criterion*. The probability distributions of the predicted two-point discrimination thresholds of our current spread model, which spanned a 95% confidence interval of 1,908–2,750 μm, was consistent with predicted thresholds for each participant when axonal stimulation was minimized, (S1 = 2,151, S2 = 2,812, S3 = 1,942 μm).

Figure 10C also shows the predicted probability distribution of the two-point discrimination thresholds after having either set θ_*rd*_ = 0 (blue curves), θ_*lift*_ = 0 (pink curves), or both θ_*lift*_ = 0 and θ_*rd*_ = 0 (yellow curves). Median values across successful parametrizations are shown with error bars representing the interquartile range of simulation outcomes in Figure 7B.

According to our model, when θ_*lift*_ = 0, the median two-point discrimination limit fell from 2,399 μm/8.3°to 2,176 μm/7.6°, pink bar and curve in Figures 7B, 10C respectively. Thus, lift might have limited the ability to spatially resolve individual electrodes in our participants.

In contrast, the effect of retinal damage (θ*_*rd*_*, = 0, blue bar and curves in Figures 7B, 10C) on two-point discrimination thresholds was very small. Although our model includes the effect that retinal damage requires higher current amplitudes to reach threshold in our model dip is calculated based on current amplitude on the retina. It is assumed, based on previous data (Greenwald et al., 2009; Nanduri et al., 2012), that brightness is linearly related to current. Since the effects of increasing current amplitude is simply to multiplicatively scale current on the retina, increases in current amplitude have no effect on *dip*. Thus, our simulations suggest that the correlation between low thresholds and better two-point discrimination found in our participants is likely “driven” by lift rather than retinal damage.

The yellow curve of θ_*lift*_ = 0 and θ_*rd*_ = 0, overlapping with the pink curve, can be thought of as a theoretical two-point resolution limit for the Argus II array if axonal stimulation, retinal damage and lift were not a factor: corresponding to an improvement of ∼0.8°.

## Discussion

As described in the Introduction, one of the main obstacles to the development of retinal prosthesis technology is that for many participants current amplitude thresholds tend to be relatively high across many or all electrodes, and only a minority of participants implanted with the Argus II clearly demonstrate pattern vision. Our aim was to understand what limits both sensitivity (perceptual thresholds) and the ability to spatially resolve two electrodes (two-point discrimination) in patients implanted with Argus II prostheses.

We measured perceptual detection thresholds, two-point discrimination thresholds, and collected self-reported daily use data. S1, who had a median amplitude threshold of 274 μA and two-point discrimination threshold of 2,394 μm, used the device most consistently. S3, who had a median amplitude threshold of 210 μA and two-point discrimination threshold of 2,161 μm, used the device averaging about 2 h a day. S2, who had a median amplitude threshold of 476 μA and two-point discrimination threshold of 3,136 μm, reported using the device once a month. While we cannot draw conclusions generalizable to larger population from a three-participant study, it is intriguing that patients S1 and S3, who had lower amplitude and two-point discrimination thresholds, used their devices far more often than S2, suggesting that two-point discrimination thresholds and or current amplitude thresholds are related to the functional utility of the device. Further research using two-point discrimination thresholds in larger cohort studies would be needed to establish the importance of this measure as a predictor of device usability in daily life functions.

Ultimately, according to our modeling, without axonal stimulation or lift our participants’ spatial resolution performance would likely have improved by ∼1.6–1.8°, or approximately 20%. Our simulated lower limit was approximately 2,176 μm/7.6°, (interquartile range 1,937–2,718 μm), equal to a spacing of almost 4 electrodes, corresponding to a logMAR acuity of roughly 2.7. This theoretical limit based on our simulations, is very similar to those observed in the better performing Argus II participants (Humayun et al., 2012; da Cruz et al., 2016).

There are a variety of reasons why a pair of electrodes might merge into a single percept.

### Current field overlap and lift

One obvious reason is overlap in electrode current fields, as demonstrated in Figure 9. This overlap is primarily driven by the physical distance between electrodes on the retinal surface and the lift of the array from the retinal surface.

Threshold amplitude has previously been shown to be correlated with electrode-retina distance (de Balthasar et al., 2008; Ahuja et al., 2013; Shivdasani et al., 2014; Xu et al., 2021). However, our simulations suggest that current threshold should not be considered a simple proxy for electrode-retina distance, since retinal damage may also play a significant role in elevating thresholds.

### Receptive field overlap

A second way percepts can overlap (also consistent with the “scoreboard model”) is when the phosphenes elicited by individual ganglion cells overlap. For the Argus II, receptive field sizes are small relative to the resolution of the array. The edge-to-edge separation of electrodes in the Argus II is approximately 1.3 degrees of visual angle. The Argus II is typically implanted over the fovea, and subtends 20° of visual angle along its longer side. At 7 degrees eccentricity (2,000 μm from the fovea), most receptive field sizes are less than 1/3°, while at 15 degrees eccentricity (∼4,300 μm from the fovea) most receptive field sizes are less than 1° (Dacey and Petersen, 1992). Thus, the loss of resolution caused by ganglion receptive field sizes was likely negligible, compared to the resolution of the array, and was not examined in our analysis. Theoretically, retinal degeneration might lead to an increase in receptive field sizes: either due to some sort of perceptual adaption, or due to a sampling bias if ganglion cells with small receptive fields were differentially affected by disease. However, this effect would have to be unrealistically massive to have any effect on spatial resolution in our participants.

### Axonal stimulation

Percepts can also overlap as a consequence of axonal stimulation, when overlapping axon fiber bundles pass under, or close to, both electrodes in a pair. We found that a “scoreboard + axon map” regression model, that included a factor based on axonal stimulation, outperformed the simple “scoreboard” (limited to amplitude and Euclidean distance) model, suggesting that axonal stimulation did play a role in reducing the ability to resolve individual electrodes. However, as shown in Figures 7, 8, the effects of axonal stimulation on two-point discrimination performance were not particularly large: a regression analysis suggested that minimizing axonal stimulation would reduce the two-point discrimination threshold by approximately 1 degree.

We did not model the effects of axonal stimulation on amplitude thresholds. However, axonal stimulation is unlikely to affect current amplitude thresholds significantly—under most stimulation protocols axonal thresholds are very similar to thresholds near the ganglion soma (Jensen et al., 2005; Vilkhu et al., 2021).

### Retinal damage

As described in the Stage III modeling section, various types of retinal damage have been observed in Argus II patients (Gregori et al., 2018; Lin et al., 2019; Rizzo et al., 2019; Patelli et al., 2020). It has been previously noted that some forms of damage such as inflammation reduces the separation between the electrodes and the retina; leading the researchers to hypothesize that this effect might serve to reduce perceptual thresholds (Gregori et al., 2018; Rizzo et al., 2019). However, this has never been confirmed with behavioral sensitivity data.

Electrical resistance is likely to be influenced by electrode-retina distance and retinal damage in complex ways. Histopathological assessments of one post-mortem implanted eye suggests the formation of fibrosis and schisis consisting of compact collagen-rich membrane with macrophages (Rizzo et al., 2019; Patelli et al., 2020). On the one hand, the vitreous fluid has low resistance, and inflammation and IRF cysts are also likely to lower resistance; on the other, membranes and fibrosis are likely to increase resistance. We did not explicitly model these interactive effects, choosing instead to simulate a wide range of *a* and *k* values.

Our simulations suggest that retinal damage may well play a significant role in elevating thresholds. However, according to our simulations (if we are correct in our assumption that brightness scales roughly linearly with amplitude) the main impact of retinal damage is high thresholds, rather than a loss of the ability to resolve individual electrodes, since our dip calculation is unaffected by a linear scaling of retinal current amplitudes.

### Strengths and limitations of our two-point discrimination paradigm

Although stimulating electrodes at double threshold amplitude was used to roughly match the percept brightness across electrodes, there likely remained significant differences in the brightness across electrodes (Greenwald et al., 2009; Nanduri et al., 2012). The percepts elicited by individual electrodes also likely differed dramatically in their shapes across the array (Luo et al., 2016; Beyeler et al., 2019). Percepts elicited by two-electrode stimulation were also likely to have been consistently brighter than single-electrode stimulation. Because many electrodes had stimulation levels near the safety limit during the experiment, it was impossible to increase stimulation amplitude on single electrodes as a means of preventing patients from using brightness as a cue (Ayton et al., 2020a). Nor was there any way of minimizing differences in percept size between single and paired stimulation.

As a result, it is likely that stimulation from single vs. paired electrodes would have produced distinguishable percepts. Our goal was to prevent participants from using brightness, shape or size information when making their “one vs. two percept” judgments.

Participants were explicitly asked to report, *“How many percepts did you see?”* and were told that brightness and the size of percepts would not provide a reliable cue. Participants reported one vs. two percepts with roughly equal frequency throughout the experiment. Importantly, their drawings (whether they drew one or two shapes) matched these verbal responses on a trial-by-trial basis. We also chose not to use single-electrode stimulation as catch trials, and gave no feedback.

However, results from this protocol should be interpreted very differently from those using a more traditional two-point discrimination methodology with single electrode catch trials and feedback (Ayton et al., 2020a). With feedback our participants would likely have quickly learned to discriminate single and dual electrode stimulation simply based on the shape and/or brightness of percepts.

### Comparison with previous studies – amplitude thresholds

Although we did not formally measure thresholds on all electrodes due to time constraints, we found that a significant proportion of individual electrodes did not elicit phosphenes using the SwiftPA procedure.

On the whole, the electrode sensitivity of our subject group seems comparable to that reported in other studies. In a previous study by Ahuja et al. (2013), detection thresholds could not be estimated within the range of amplitudes permitted by charge density safety limits in a significant proportion of electrodes (0–83% depending on participant). In a study by Naidu et al. (2020), thresholds could only be measured in 60% of electrodes. Xu et al. (2021) similarly could not measure individual thresholds in a significant proportion of electrodes.

### Comparison with previous studies – spatial vision

As shown in Table 7, grating acuity, direction of motion discrimination, and square localization are the most commonly used measures of the spatial resolution of the Argus II implant. While these tasks provide a good assessment of real-world spatial acuity, they are influenced by both eye and head-movements, and therefore cannot be used to measure losses in spatial resolution at the retinal level, which is best assessed by two-point discrimination task. A previous study has found a correlation between two-point discrimination and grating spatial acuity in Argus II patients (Lauritzen et al., 2011), suggesting that resolution at the retinal level does influence visual performance on other tasks that are more closely related to “real world” vision.

**TABLE 7:** Comparison of tasks used to measure spatial acuity.

Task	Within-array resolution required	Affected by eye- and head movements	Literature
Two-point resolution	Yes*	No	Lauritzen et al., 2011
Grating acuity	Yes*, at frequencies above 2.9 logMAR in the Argus II (Stronks and Dagnelie, 2014)	Yes	Better than 2.9 logMAR Humayun et al., 2012: 21.88% Ho et al., 2015: 48.2–33.3% da Cruz et al., 2016: 38% Schaffrath et al., 2019: 10% Arevalo et al., 2021: 40%
Square localization / Direction of motion	Within array localization is not required for square localization or direction of motion (with feedback). For square localization (and possibly direction of motion) it is likely that many participants rely on scanning head-movements and use the percepts generated by the array as a merged single “phosphene” (Peli, 2020).	Yes	Ahuja et al., 2011; Humayun et al., 2012; Rizzo et al., 2014; Ho et al., 2015; da Cruz et al., 2016; Schaffrath et al., 2019; Naidu et al., 2020; Arevalo et al., 2021

*As described above, cues such as brightness and shape distortions are extremely difficult to entirely eliminate in Argus II participants.

Table 7 summarizes previous studies that assessed spatial vision with the Argus II across a range of tasks.

Out of our nine original participants only three showed evidence of within array resolution and were selected for further testing. Although other studies have not examined two-point discrimination, the grating acuity task at spatial frequencies higher than 2.9 logMAR also requires within-array resolution (though this task may be more difficult, due to blurring due to eye-movement/head motion). In previous studies only 10–40% of Argus II patients performed better with the device on vs. off in a grating acuity task at frequencies higher than 2.9 logMAR, Table 7.

In previous studies it has been difficult to find a clear link between either height from the retinal surface or retinal damage as measured using OCT and spatial performance (Rizzo et al., 2019). One reason for this may be that many tasks used for functional assessment (e.g., square localization) are not specifically designed to test within-array resolution while excluding the effects of eye-movements.

### Limitations

One important limitation of our study is that we collected two-point discrimination data in just three participants, those tested at Johns Hopkins Eye Center. Moreover, as noted in the Methods, these three were selected as the best of 9 participants across two centers. Such a small participant group cannot support population level inferences; our data are best considered as three “case studies” illustrating a range of outcomes. In addition, because our data were collected over a relatively small number of sessions we do not have longitudinal data that might provide insight into the effects of the array shifting/lifting or continued retinal degeneration.

Our simulations also include significant uncertainty. First, our estimates of the distance to axon certainly includes variability due to errors in our estimation of axon bundle trajectories. Second, our estimates of current spread include a broad range of possible values, making our estimates of the relative importance of electrode lift and retinal damage quite broad. Finally, the_θ_*rd*_ parameter that we interpret as retinal damage, simply reflects an increase in threshold unexplained by_θ_*lift*__, which potentially could be explained by other factors.

### Future directions

Placing an electrode array close to the surface without causing retinal damage is extremely difficult (Gregori et al., 2018). This makes it important to know whether successful outcomes depend on placing an array proximal to the retinal surface, avoiding retinal damage or (more likely) both. Unfortunately, we could not obtain high quality OCT images that would allow us to directly estimate the height of electrodes from the retinal surface, so our simulations can only indirectly infer the relative importance of retinal lift vs. retinal damage. However, our simulations do suggest, somewhat unsurprisingly, that avoiding both lift and significant retinal damage are likely to critical for a successful retinal implant.

Future work relating two-point discrimination to imaging data that includes array-retina positioning, structural measures of retinal integrity and more detailed computational modeling, based on data from a larger number of participants will likely be needed to fully understand the relative importance of these various factors in reducing the ability to resolve the percepts elicited by individual electrodes, and thereby develop implants which can successfully subserve pattern vision.

## Data availability statement

The datasets presented in this study can be found in online repositories. The names of the repository/repositories and accession number(s) can be found below: https://github.com/VisCog/Argus_current_spread.

## Ethics statement

The studies involving human participants were reviewed and approved by University of Washington and Johns Hopkins University, Institutional Review Boards. The patients/participants provided their written informed consent to participate in this study. Written informed consent was obtained from the individual(s) for the publication of any potentially identifiable images or data included in this article.

## Author contributions

EY, MB, IF, and AR contributed to the conception and design of the study. EY, MB, AK, RS, SM, GB, and GD collected the data. EY, AR, GB, and IF performed the modeling and statistical analysis. EY and IF wrote the first draft of the manuscript. All authors contributed to the data and modeling interpretation, manuscript revision, read, and approved the submitted version.
